# Primary care nurses struggle with lifestyle counseling in diabetes care: a qualitative analysis

**DOI:** 10.1186/1471-2296-11-41

**Published:** 2010-05-25

**Authors:** Renate Jansink, Jozé Braspenning, Trudy van der Weijden, Glyn Elwyn, Richard Grol

**Affiliations:** 1 Scientific Institute for Quality of Healthcare, Radboud University Nijmegen Medical Centre, P.O. Box 9101, 6500 HB Nijmegen, The Netherlands; 2 Department of General Practice, School for Primary Care and Public Health, Maastricht University, P.O. Box 616, 6200 MD Maastricht, The Netherlands; 3 Department of Primary Care and Public Health, School of Medicine, Cardiff University, Heath Park CF14 4XN, UK

## Abstract

**Background:**

Patient outcomes are poorly affected by lifestyle advice in general practice. Promoting lifestyle behavior change require that nurses shift from simple advice giving to a more counseling-based approach. The current study examines which barriers nurses encounter in lifestyle counseling to patients with type 2 diabetes. Based on this information we will develop an implementation strategy to improve lifestyle behavior change in general practice.

**Method:**

In a qualitative semi-structured study, twelve in-depth interviews took place with nurses in Dutch general practices involved in diabetes care. Specific barriers in counseling patients with type 2 diabetes about diet, physical activity, and smoking cessation were addressed. The nurses were invited to reflect on barriers at the patient and practice levels, but mainly on their own roles as counselors. All interviews were audio-recorded and transcribed. The data were analyzed with the aid of a predetermined framework.

**Results:**

Nurses felt most barriers on the level of the patient; patients had limited knowledge of a healthy lifestyle and limited insight into their own behavior, and they lacked the motivation to modify their lifestyles or the discipline to maintain an improved lifestyle. Furthermore, nurses reported lack of counseling skills and insufficient time as barriers in effective lifestyle counseling.

**Conclusions:**

The traditional health education approach is still predominant in primary care of patients with type 2 diabetes. An implementation strategy based on motivational interviewing can help to overcome 'jumping ahead of the patient' and promotes skills in lifestyle behavioral change. We will train our nurses in agenda setting to structure the consultation based on prioritizing the behavior change and will help them to develop social maps that contain information on local exercise programs.

## Background

Despite the apparent advantages of behavioral risk factor management for patients with type 2 diabetes, the predominant focus in primary care is still on curative care, and the transition is taking place at a slow rate [1]. Providing preventive service in general practice is often delegated to primary care nurses. In the Netherlands about 80% of the practices have a primary care nurse [2]. All patients in general practice can be admitted to this nurse, because health insurance companies take the cost. However, we know that patient outcomes on lifestyle advice (diet, physical activity, smoking) in general practice are poor [3-5]. The complexities of changing behavior requires nurses to shift from simple advice giving, as described in most diabetes guidelines, to a more counseling-based approach [1]. Behavioral risk factor management attempts to inform the patient about diabetes and the relation of diabetes complications to diet, physical activity, and smoking behavior. At the same time, the patient must become motivated to change his/her lifestyle and become a believer in his/her own abilities. The primary care nurse tries to promote behavioral change in a supportive, empathic, and comprehensive way, and in the meantime, she must see to it that practical barriers are overcome to facilitate the behavioral change of the patient [6]. These activities take place in the limited time span of a quarterly check-up appointment that, in the Netherlands, takes usually 15 to 20 minutes. In addition, during this time, the glucose level, blood pressure, and weight must be measured, and information about the effect of the medication is updated. Not surprisingly, some studies suggest that nurses lack time and skills to promote lifestyle changes and risk reduction [7-11]. However, the success of the lifestyle counseling depends not only on the nurse's efforts, but also on the patient's open mind and perseverance, as well as the conditions of the practice [12]. The aim of this study was to gain insight into the lifestyle counseling barriers that nurses encounter on these three levels (nurse, patient, practice). The results will be used to develop an implementation strategy to improve lifestyle behavior change for patients with type 2 diabetes in general practice. The implementation strategy will be used in a randomized controlled trial (RCT), the MILD study [13].

## Methods

### Study design

Semi-structured, in-depth interviews with nurses were conducted to help us better understand the specific barriers of lifestyle counseling in general practice and to help us to design and develop a more effective program for the MILD study. The nurses from the first 12 of the 70 practices that participated in the randomized controlled MILD trial [13] were invited by telephone for an interview before the trial. If more than one primary care nurse was employed in a practice, only one of them was interviewed. Each interview was conducted in the general practice of the nurse. The interviewer examined the 12 interviews to determine whether content saturation had been achieved. If saturation had *not *been achieved, additional interviews would have been scheduled. The medical Ethics Committee of the University of Nijmegen has granted ethical approval (16 January 2006; Reference number JvG/CMO 0116).

### Data collection

The interview questions included a combination of prestructured and open-ended questions (Table 1). The nurses were asked to describe the barriers they encountered at the three levels (i.e., the nurse, patient, and practice levels) during counseling regarding the provision of diet, physical activity, and smoking cessation. The research team discussed the precise formulation of the questions until mutual agreement was achieved. The first author (RJ) guided the discussion and used a checklist to make sure that all potential barriers were adequately discussed. The same person conducted all of the interviews, which were audio-recorded.

**Table 1:** Interview questions

*Broad questions*	What barriers do you encounter in diet counseling?
	What barriers do you encounter in physical exercise counseling?
	What barriers do you encounter in smoking cessation counseling?
*Specific questions*	
Patient level	What barriers occur at the patient level when you give diet counseling?
	What barriers occur at the patient level when you give physical exercise counseling?
	What barriers occur at the patient level when you give cessation counseling?
	
Provider level	What barriers do you encounter with skills for diet counseling?
	What barriers do you encounter with skills for physical exercise counseling?
	What barriers do you encounter with skills for cessation counseling?
	
Practice level	What barriers do you encounter at the practice level while provide lifestyle counseling (e.g., barriers with consultation time, barriers with organization of the diabetes care)?

### Procedure and data analysis

The interviews were transcribed verbatim. Data analysis were done according to the framework approach [14]. Two researchers (RJ and MdB) independently reviewed the transcripts and classified the comments according to a predetermined framework based on several theoretical reflections on behavioral change as described by Grol et al. [15]. In cases of disagreement, consensus was achieved via discussion with a third researcher (JB). In this framework, the three main levels of barriers to lifestyle counseling (the nurse level, the patient level, and the practice level) were subdivided into several categories. At the nurse level, the categories were awareness, knowledge, attitudes, motivation to change, and behavioral routines. At the patient level, the categories were knowledge, attitudes, skills, and compliance. At the practice level, the categories were organization of care processes, staff, capacities, resources, and structures [15]. Additional categories were formulated for barriers that did not fit into the categories of the original framework.

## Results

### Characteristics of primary care nurses

Table 2 summarizes the characteristics of the primary care nurses. All of the nurses initially invited to participate in the trial agreed to take part. In one general practice, we interviewed two nurses as this was preferred by the practice over an interview with only one. A total of 13 primary care nurses were interviewed.

**Table 2:** Study population

Characteristics of primary care nurses	*n *= 13
Men/women	0/13
Mean age in years (range)^1^	44 (27-51)
Primary care nurses who were formerly practice assistants (percentage)^1^	6 (50%)
Mean years of experience as practice assistant (range)^1^	12.3 (2-24)
Mean years of experience working with diabetes (range)^1^	3.0 (0.5-4.5)

^1 ^After the interviews, one nurse withdrew of the study. Her characteristics were unknown

The mean age of the nurses was 44 years (range 27 - 51 years), and all were women. They had an average of 3.0 years (range 0.5 - 4.5 years) of experience with diabetes consultation. Six of the nurses (50%) had an average of 12 years (range 2 - 24 years) of experience as practice assistants in the Netherlands. A practice assistant is somebody who supported the general practitioners and worked predominantly as receptionist and administrative assistant [16]. The participating nurses were trained as a nurse in a 3 or 4 years program (middle or higher education) and specialized in primary care for respectively another 2 or 1 year program.

### Interviews

The last two interviews added no new information. No more practices were invited for an interview because saturation had been reached. A few adjustments were made to the predetermined framework during the analysis. The categories "awareness" was omitted at the provider level because the nurses were well aware of the necessity of changing their method of lifestyle counseling that is why they enrolled in our study. At nurse level the 'motivation to change' category was substituted with 'skills' which described the responses more accurately and was congruous with the category at patient level (Table 3). At practice level, three categories were combined in order to prevent overlap (Table 4) and we subdivided categories based on the different issues raised (Tables 3, 4, 5).

**Table 3:** Barriers to lifestyle counseling in diabetes care at the nurse level

Categories	Knowledge	Attitude	Skills	Behavior routines
Subcategories	1. Lack of knowledge about diet and physical activity	1. Lack of motivation 2. Relationship with patients 3. Lack of empathy 4. Stress caused by pressure of time	1. Lack of communication skills 2. Making concrete plans in a structured manner 3. Working faster than patients	1. Involving patients in decision-making

**Table 4:** Barriers to lifestyle counseling in diabetes care at the patient level

Categories	Knowledge	Attitude	Skills	Compliance
Sub categories	1. Little insight and knowledge of one's own behavior and health 2. Language barriers 3. Wrong information from the environment (social influences)	1. Unwillingness caused by: a. Not liking to change or not wanting to change b. Age c. Previous experience with dietician 2. Excuses 3. Habits 4. Cultural differences	1. Physical restrictions 2. Financial restrictions 3. Location of exercise programs 4. Addiction to smoking 5. Noncompliance with advice 6. Psychosocial troubles	1. Lack of immediate results 2. Difficult moments 3. Potential relapse 4. Lack of discipline in maintenance

**Table 5:** Barriers to lifestyle counseling in diabetes care at the practice level

Categories	Organization of care processes, staff, and capacities	Resources	Structures
Subcategories	1. Lack of time 2. Poor cooperation between practice nurse and other health providers	1. Local exercise map missing	1. Insufficient information material 2. The stop-smoking protocol is inadequate

#### The nurse level

##### Knowledge

Some nurses reported lacking sufficient knowledge about physical activity, smoking cessation, and even more notable, about specific diet advice in order to provide adequate lifestyle counseling. They also explained that it is a dietician's task to give specific diet advice to patients. However, some patients are not willing to see a dietician. In such cases, the nurse felt a need to give the diet advice to the patient.

"Some patients have had bad experiences with dieticians and refuse to go to them. This means that I have to get down to the diet advice. I can tell the patients what is good or bad for them, but for specific diet advice they still have to go to the dietician." (N2)

The knowledge deficiency felt masks a shortage of coordination capability with dietician and of communication skills of convincing explanation to patients.

##### Attitudes

Some nurses mentioned they sometimes lack motivation themselves because they have to repeat the lifestyle message again and again, and they have little hope that the patient will change. That makes them feel very powerless. Other nurses did not like to be judgmental and were hesitant to discuss lifestyle behavior change if they thought that would put the relationship with the patient at stake. Another barrier nurses mentioned is lack of empathy. This can occur when nurses do not understand why it is so difficult to change a specific lifestyle which is not a barrier for themselves. Furthermore, they found it difficult to be patient and listen carefully when they were stressed for time.

"It is very difficult for patients to change their lifestyles. I have to tell them the same thing all the time, mostly without any result. This makes me feel powerless." (N3)

"I like to see the patients pleased to come back, so I want to have a good relationship with them. Sometimes I am too soft. This is wrong because I have to help patients change their lifestyles." (N 3)

##### Skills

Nurses repeatedly highlighted a deficiency in their lifestyle counseling skills. They do not know how to develop a concrete and structured action plan in cooperation with the patient. They also reported having difficulties in adapting their counseling to the stage in which the patient is.

"I do not know what the best way is to counsel patients. At the end of the consultation, I must have a concrete action plan, such as: eat less high-fat cheese. It is difficult to make things concrete and do this in a structured manner." (N 11)

"Sometimes I supply information too fast. The patients are in an earlier stage of change." (N 13)

It seems that the nurses are jumping ahead of the patient. Nurses had false or too high expectations for lifestyle change by patients. This results in a righting reflex where they push too hard for change, resulting in resistance of the patient. Nurses explicitly told us that they would like to have some skills to overcome this barrier.

##### Behavioral routines

General practice did not had a long history of providing preventive services to patients. For that reason, primary care nurses reported having difficulties getting rid of old or inappropriate routines. For example, the nurse had to involve patients in decision making, but in practice they regularly gave simple lifestyle information and advice to patients.

"Some patients can hardly move. I look for alternatives to solve the barrier, but I have to give these patients time to look for a solution themselves." (N 7)

#### The patient level

##### Knowledge

Nurses thought that while patients have a general feeling of the urgency to change, they lack insight into their lifestyle behavior, health, and, in particular, the effects of their diet. Since patients think they know how to live healthy, they often refuse to see a dietician. Furthermore, the nurses thought that language could be a barrier for patients from other cultures and for patients who have a low level of understanding. Misinformation from peers could also interfere with lifestyle counseling.

*"Patients do not know what carbohydrates and glucose are, or they think they eat healthful food while it is actually fatty*. *Some patients refuse to see a dietician because they think that they already know everything there is to know about diet." (N 4)*

"Patients from other cultures do not understand the lifestyle counseling because they do not master the Dutch language. However, some Dutch people do not understand the counseling either." (N 2)

"The social control over diet is very strong among people in small villages. Sometimes, diabetes is discussed at birthday parties where patients can give each other incorrect pointers about food." (N 8)

##### Attitudes

According to the nurses, the unwillingness of patients to change their lifestyle is based on a general aversion to change and previous experience with a dietician. This was seen more often among the elderly. Furthermore, nurses thought that patients search for excuses not to give up their habits.

"Patients think it is very difficult to stop smoking, but most patients who actually stopped thought afterwards that it exceeded their expectations." (N 3)

"It is difficult to motivate older patients with diabetes to eat healthful food - they say: 'I am 75 years old and I do not intend to go on a diet,' 'I have reached a good old age with my own eating habits,' or 'If I live 10 years less - so what? I'm alone anyway.'" (N 10)

"Patients with diabetes are often not used to physical activity, so it is very difficult for them to start exercising at an older age. They search for excuses: 'I have a backache', 'The weather is bad,' or 'I'm tired after a busy workday.' Any excuse not to move." (N 5)

The expressions of the patients indicate the need to patient-centeredness, but nurses did not report opportunities to start a discussion on patients motivation to change lifestyle behavior.

##### Skills

Most patients with type 2 diabetes are older people with physical disabilities and sometimes low incomes. The nurses thought that it was not easy for a patient to increase his/her level of physical activity and not possible at all when there is no gym or fitness center in the neighborhood. Furthermore, smoking is an addiction. The nurses said that sometimes patients try to quit smoking by cutting back, but quickly relapse. Patients with psychosocial barriers often do not want to stop smoking, according to the nurses.

"Patients with physical restrictions hardly exercise, even if they want to; they cannot afford to go to a gym. It is very expensive." (N 1)

"People have to travel considerable distances because there is no gym or exercise club in their neighborhood." (N 9)

##### Compliance

The compliance-related barriers that the nurses mentioned are lack of immediate results, lack of discipline for maintenance, potential for relapse, and difficult moments such as stress situations and situations in which other people lure patients into unhealthy behaviors.

"A change of lifestyle does not immediately lead to positive results. Patients find it difficult to deal with these challenges and gradually lose heart, which often results in a relapse to an unhealthy lifestyle." (N 13)

#### The practice level

##### Organization of care processes, staff, and capacities

Some nurses reported lacking sufficient time for additional tasks such as keeping pace with new developments. Practice management will not provide more time because it is expensive. The cooperation between health providers was good in most cases, but some nurses felt that they did not get enough feedback from the dietician. There was also a lack of clarity with regard to roles and responsibilities between the nurses and dieticians. The nurses sometimes simply told us, for instance, that diet counseling is the dietician's task. A much-needed consultation with the general practitioner was also sometimes skipped because of an emergency.

"There is not enough consultation with the dietician. I often do not know what kind of diet arrangement has been made with the patient. Or sometimes the general practitioner has to attend to an emergency and our conversation is left unfinished." (N 2)

##### Resources

A so-called local exercise file is an overview of local sport schools and other physical activity facilities in the area. Exercise files were not available in some of the general practices resulting in nurses having little knowledge of the local exercise programs for patients with diabetes. They also did not know whether reimbursement for the exercise programs was possible.

##### Structures

Nurses told us that they lack high-quality patient education materials for effective lifestyle counseling. In addition, some nurses found the protocol used for smoking cessation inadequate for optimal counseling. They have heard from colleagues that there is a better protocol, but the course is very expensive - too expensive for their general practice.

## Discussion

Nurses perceived considerable barriers to lifestyle counseling in general practice. We looked separately to the three areas of lifestyle (diet, physical activity, and alcohol consumption), but did not see differences in barriers named. So we decided to focus on the general level. There was significant agreement between the nurses on the barriers reported. As expected, barriers include insufficient time and lack on counseling skills [7-11]. However, most barriers mentioned by nurses were on the level of the patient. Nurses reported that patients have limited knowledge of a healthy lifestyle and limited insight into their own behavior. Moreover, they have little motivation to modify their lifestyles or the discipline needed to maintain an improved lifestyle.

The results of the current study are consistent with previous research that explores the barriers that healthcare providers encounter during patient counseling. Considering the barriers at the patient level, general practitioners mention the excuses that patients use to avoid having to change poor health habits [11]. Other healthcare providers also frequently report difficulties with patient motivation and unwillingness to change [4,9,11,17,18]. It is important that the providers of lifestyle counseling be familiar with the patients' barriers [4,11,19,20]. The participating nurses in our study are well informed about which barriers much be confronted during the provision of lifestyle counseling. The nurses were very willing to help patients with regard to lifestyle changes and considered it an important part of the care for patients. Nonetheless, it is difficult to deal with certain patient barriers, and nurses sometimes feel powerless when patients do not attain the goals that have been set. This may frustrate both patients and nurses, reduce the nurses' empathy for at least some patients, and reduce their motivation to counsel patients in general. This is in line with research showing that general practitioners who try to provide good diabetes care via the presentation of evidence-based recommendations experience considerable frustration when patients do not adopt or adhere to these recommendation [11]. It is very difficult to convince patients of the necessity of lifestyle changes. Nurses may have to motivate patients to change, but are not prepared for this task. Furthermore, when possible solutions to overcome these barriers are discussed, nurses suggested that extra training in counseling skills might help them motivate patients. Primary care providers in other studies also report a lack of skills for facilitating behavioral changes as a barrier at the nurse level [11,21-23]. The nurses interviewed for this study had a specific education for general practice. They were trained in counseling techniques. However, training in communication techniques alone is not enough to change lifestyle behavior. Karhila et al. suggest that nurses might need to become more aware of their counseling practices in routine situations through conscious effort for self-evaluation [24].

Professionals and patients in general practice have indicated a preference for a more patient-centered approach [25]. However, changing from health education is not an easy task. Allowing patients the freedom to make their own decisions about their health behaviors can be a difficult task for nurses [24]. Nurses feel a professional responsibility to accurately advise the patient rather than allow the patient to make a decision for themselves. Ineffective lifestyle counseling was also attributed to lack of time for the nurses providing such counseling, which is a well-known barrier at practice level [26,27]. The nurses told us that, due to this lack of time and insufficient knowledge of diet counseling, they could not really address the patient's diet [28]. However, in some studies a majority of professionals reported little or no increase in time demands [29]. Furthermore, some nurses thought that diet counseling was not their task but, rather, the dietician's task. Given that the cooperation between dietician and nurse is not optimal, patients are then left with, possibly critical, diet questions and a lack of help to improve their diets.

The most important finding is that the nurses notify a lot of barriers on the patient level. This can be explained by two factors. First, nurse tend to have false or too high expectations for lifestyle change by their patients (jumping ahead of the patient), resulting in resistance of the patient. Secondly, the provision of simple lifestyle information and advice was the predominant strategy used by primary care nurses, as evidence by the contradictions in the nurses' language use: 'counseling' versus 'I have tell them the same thing all the time', 'advice', 'sometimes I'm too soft', 'supplying information'. While advice giving is an important task in lifestyle counseling, it is associated with increased patient resistance if unsolicited. Lifestyle counseling that can help to overcome these processes is motivational interviewing. Motivational Interviewing is a patient-centered, directive method for enhancing intrinsic motivation to change by exploring and resolving ambivalence [30]. Research has shown that motivational interviewing is an effective strategy in implementing recommendations on lifestyle [7,31]. This strategy should focus on brief consultations and seems to be a great opportunity to solve many barriers mentioned.

Two methodological issues need to be addressed. First, as the nurses joined the study voluntarily, it might be that they are not a representative sample of the general population of nurses. Thus there may be limits to generalizing these findings. However, findings from other studies suggest that most providers of care experience similar barriers [11,18,21,32]. Second, it was sometimes difficult to fit the data into the framework. Primary care nurses mentioned the "absence of a local exercise file" and "unsuitable leaflets regarding lifestyle recommendations" as barriers at the practice level. These, however, could also be classified as a "shortage of knowledge" at the level of the nurse. Our strategy was to classify them according to the category that the nurses mentioned.

## Conclusions

The traditional health education approach is still predominant in primary care of patients with type 2 diabetes. Nurses were often inclined to take over the responsibilities of the patient too quickly, while the lifestyle guidance of patients with diabetes is more effective when the responsibility is shared. The nurses also found it difficult to motivate patients, and reported a need to improve their own counseling skills. What nurses require are concrete tools to increase patient adherence to recommended lifestyle changes and ways to build these tools into daily care. Given the limited proportion of patients who actually change their behavior, lifestyle counseling emerges as an unrewarding part of the primary care nurse's job.

Based on our results we will develop an implementation strategy, built on motivational interviewing, that can help to overcome 'jumping ahead of the patient' and promotes skills in counseling for lifestyle behavioral change. We also make room for agenda setting of the patient to structure the consultation based on prioritizing the necessary behavior change. We will train our nurses in motivational interviewing as well as agenda setting and help them with developing social maps that contain information on local exercise programs.

## Competing interests

The authors declare that they have no competing interests.

## Authors' contributions

RJ, the main investigator, carried out the qualitative research. She conducted all semi-structured in-depth interviews with primary care nurses, analyzed the interview transcripts and drafted the manuscript. JB, the project leader, involved in all aspects of the study. TvdW, GE, and RG participated in discussions about the analyses of the study and the reporting. All authors read and approved the final version of the manuscript.

## Pre-publication history

The pre-publication history for this paper can be accessed here:

http://www.biomedcentral.com/1471-2296/11/41/prepub
